# Delayed Transplantation of Neural Stem Cells Improves Initial Graft Survival after Stroke

**DOI:** 10.1002/advs.202504154

**Published:** 2025-05-23

**Authors:** Rebecca Z Weber, Nora H Rentsch, Beatriz Achón Buil, Melanie Generali, Lina R. Nih, Christian Tackenberg, Ruslan Rust

**Affiliations:** ^1^ Institute for Regenerative Medicine University of Zurich Schlieren 8952 Switzerland; ^2^ Neuroscience Center Zurich ETH Zurich and University of Zurich Zurich 8057 Switzerland; ^3^ Department of Brain Health Kirk Kerkorian School of Medicine University of Nevada Las Vegas NV 89154 USA; ^4^ Department of Physiology and Neuroscience University of Southern California Los Angeles CA 90033 USA; ^5^ Zilkha Neurogenetic Institute Keck School of Medicine University of Southern California Los Angeles CA 90033 USA

**Keywords:** cell therapy, iPSCs, ischemia, NPC, NSC, stem cells, stroke

## Abstract

Neural stem cell therapies hold great promise for improving stroke recovery, but the hostile stroke microenvironment can hinder the initial graft survival. It has long been well documented that the microenvironment evolves over time, making it crucial to identify the optimal transplantation window to maximize therapeutic efficacy. However, it remains uncertain whether acute or delayed local cell transplantations better supports graft viability after stroke. Here, it is shown that delayed intracerebral transplantation of neural progenitor cells (NPCs) derived from human induced pluripotent cells (iPSCs) at 7 days post stroke significantly enhances graft proliferation and survival, and promotes axonal sprouting, compared to acute transplantation at 1 day post stroke, in a mouse model of large cortical stroke. Using in vivo bioluminescence imaging over a 6‐week period post‐transplantation, a more than fivefold increase is observed in bioluminescence signal in mice that received delayed NPC therapy, compared to those that underwent acute NPC transplantation. The increased number of cell grafts in mice receiving delayed NPC transplantation is driven by increased proliferation rates early after transplantation, which subsequently declines to similarly low levels in both groups. Notably, it is found that the majority of transplanted NPCs differentiate into neurons after 6 weeks, with no significant differences in the neuron‐to‐glia ratio between acute and delayed transplantation groups. These findings suggest that delayed NPC transplantation improves early graft survival and proliferation, which could help identify the optimal therapeutic window for maximizing the effectiveness of NPC‐based therapies in stroke.

## Graphical abstract

Delayed transplantation of human iPSC‐derived neural progenitor cells (NPCs) enhances graft survival, proliferation, and axonal sprouting after stroke. Using bioluminescence imaging and histology in a mouse model of stroke, it is shown that NPC delivery at 7 days post‐stroke is more effective than acute transplantation, highlighting the importance of timing for maximizing therapeutic efficacy in cell‐based stroke therapies.

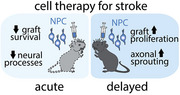

## 1 Introduction

Stroke remains a leading cause of disability and death worldwide with no regenerative treatments available.^[^ ^1^ ^]^ Current acute treatments, such as intravenous thrombolysis (tPA) and mechanical thrombectomy, must be administered within a critical short time window and are not applicable for many patients.^[^ ^2^, ^3^ ^]^ Stem cell therapy is considered one of the most promising experimental strategies for enhancing stroke recovery beyond this acute window, as transplanted cells have the potential to regenerate lost brain tissue and promote functional repair.^[^ ^2^ ^]^ A promising cell source for cell therapy are neural progenitor cells (NPCs) derived from induced pluripotent stem cells (iPSCs), as they can be expanded indefinitely and differentiated into any cell type, including mature neurons, and glia as shown by us and others previously.^[^ ^4^ ^]^ Although local brain transplantation of NPCs has shown preclinical efficacy, the optimal timepoint for transplantation after stroke remains unclear.^[^ ^5^ ^]^ While some studies suggest that delayed cell transplantation could be beneficial to avoid the acute inflammatory stroke environment and associated poor cell survival,^[^ ^6^, ^7^ ^]^ other preclinical and clinical research suggests that cell‐based therapies may be most effective when administered as early as 24 h after stroke onset.^[^ ^8^, ^9^, ^10^ ^]^

We hypothesized that delaying NPC transplantation would enhance survival and tissue integration compared to acute transplantation. To test this hypothesis, we locally transplanted NPCs into mice with large cortical strokes at acute (1‐day post‐injury, dpi) and delayed (7 dpi) timepoints. Over a 43‐day period post‐transplantation, we found that NPCs transplanted at 7 dpi displayed higher proliferation and survival rates. Specifically, the proliferation rate was notably higher in the early days following transplantation, suggesting that the hostile stroke environment limits the acute growth of NPCs. Furthermore, we observed that most transplanted cell grafts successfully differentiated into mature neurons and glia within six weeks. The neuron‐to‐glia ratio remained consistent across both acute and delayed transplantation groups, indicating no significant effect of transplantation timing on NPC fate commitment.

## 2 Results

### 2.1 Delayed NPC Transplantation Leads to a Higher Detectable Graft Signal over Six Weeks

**Figure 1:**
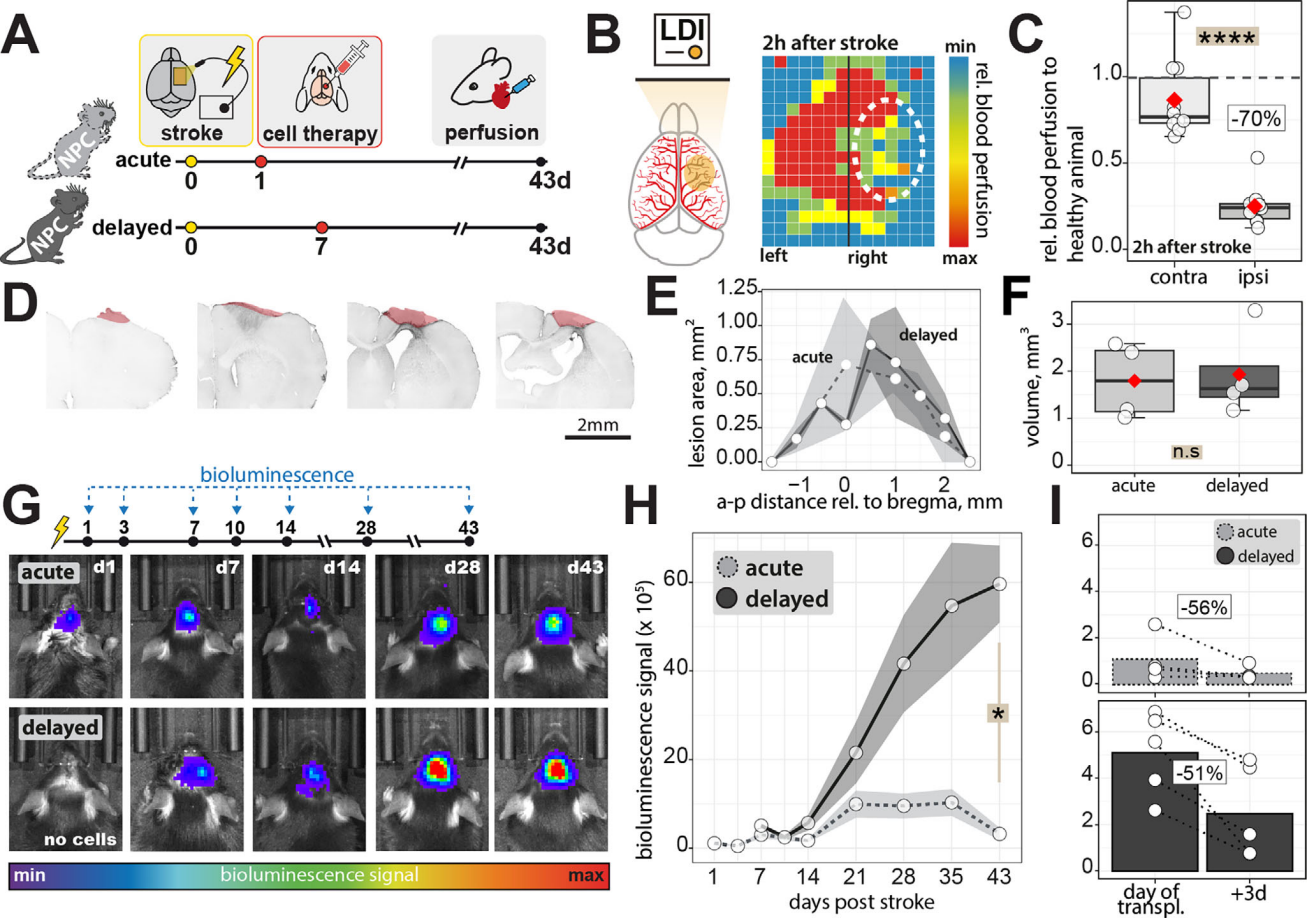
Delayed transplantation of NPCs improves long‐term graft survival post‐stroke. A) Schematic illustration showing the experimental design. Immunodeficient Rag2^−/−^ mice underwent stroke induction followed by local transplantation of rFluc‐expressing NPCs at either 1 dpi (acute) or 7 dpi (delayed). B) Laser Doppler imaging confirms reduced cerebral blood flow (CBF) after stroke. C) Quantification of CBF 2 h after stroke induction. D) Coronal brain sections used to estimate stroke area. The dotted red line indicates the stroke‐lesioned brain area. Scale bar: 2 mm. Stained with GFAP antibody. E) Quantification of stroke infarction size. Lesion area plotted against the anterior‐posterior (a‐p) distance relative to bregma (mm) and F) boxplot displaying lesion volumes (mm^3^) of both treatment groups. G) Representative bioluminescence imaging (BLI) illustrating NPC survival over 6 weeks in both groups of selected time points. H) Quantification of BLI signal over time and I) within the first 3 days after transplantation in both groups. Data are shown as mean distributions where the red dot represents the mean. In (C, H, and I) a total of N = 10 animals were used, with 5 animals per group; in (E, F), a total of N = 8 animals were used, with 4 animals per group; Boxplots indicate the 25% to 75% quartiles of the data. For boxplots: each dot in the plot represents one animal. Line graphs are plotted as mean ± SEM. Significance of mean differences was assessed using an unpaired Mann‐Whitney U Test (C, F, H). Statistical significance was set at *p < 0.05; **p < 0.01; ***p < 0.001.

To determine the survival and proliferation of the cell grafts, we locally transplanted NPCs expressing firefly luciferase (rFluc) in immunodeficient Rag2^−/−^ mice at 1 day (NPC_acute_) or 7 days (NPC_delayed_) post‐stroke (**Figure** **1**A). NPCs were differentiated from human iPSCs under transgene‐ and xeno‐free conditions.^[^ ^11^ ^]^ This cell source has been extensively characterized to spontaneously differentiate into astrocytes and functional, mature neurons and is compatible with safety switches for in vivo applications.^[^ ^11^ ^]^ For this study, we verified the expression of canonical NPC marker Nestin while pluripotency marker Nanog was absent in the NPC population (Figure S1A,B, Supporting Information**)**.

We first confirmed successful stroke induction across all mice, resulting in at least 50% reduction in cerebral blood flow 2 h after induction compared to the contralesional site (p < 0.0001) (Figure 1B,C). Mice were then randomly allocated to experimental groups with a similar stroke severity across groups. 43 days after stroke induction, infarcted tissue extended from ‐2 to +2.5 mm anterior‐posterior relative to bregma (Figure 1D,E). Lesion volumes were comparable between both groups (NPC_acute_: 1.8 ± 0.4 mm^3^, NPC_delayed_: 1.93 ± 0.47 mm^3^, p = 0.8, Figure 1F).

Next, we analyzed long‐term survival of rFluc‐expressing NPC_acute_ and NPC_delayed_ throughout the time course of 6 weeks after stroke via bioluminescence imaging (Figure 1G). After 43 days post stroke, we observed a considerably stronger bioluminescence signal (NPC_acute_: 3.2 ph/s/cm3/sr x 10^5^) vs NPC_delayed_: 60 ph/s/cm3/sr x 10^5^, p < 0.05) (Figure 1H). Additionally, a notable decrease in bioluminescence signal was observed within the first 3 days after transplantation, with a reduction of 56% in the NPC_acute_ group compared to 51% in the NPC_delayed_ group (Figure 1I). Overall, these findings suggest that delayed NPC transplantation results in a higher detectable graft signal over six weeks, indicating potential benefits for graft survival and proliferation after stroke.

### 2.2 Delayed NPC Transplantation does Not Affect Vascular Remodeling, Glial Scarring, or Apoptosis

**Figure 2:**
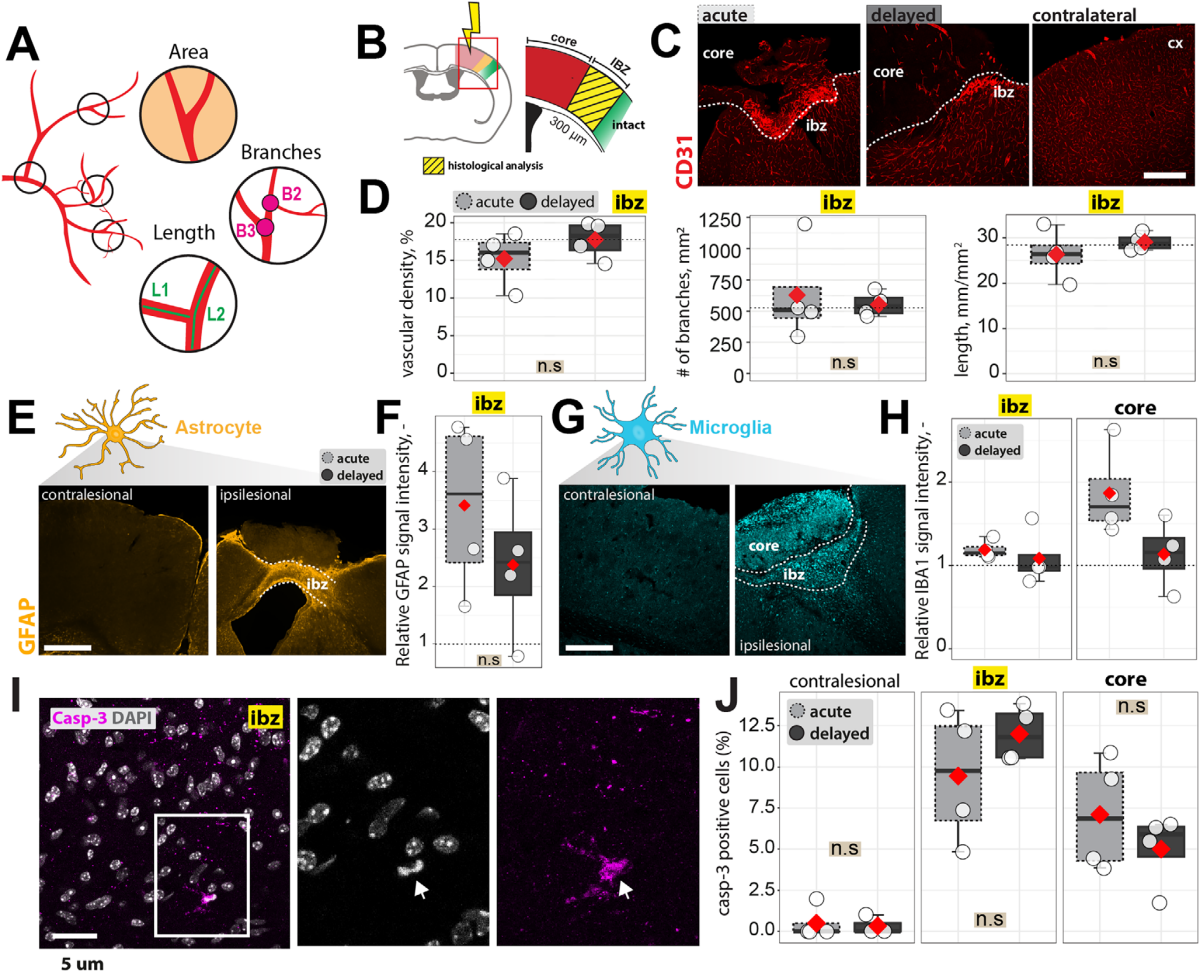
Anatomical changes in ischemic stroke tissue in acute and delayed transplantation groups. A) Representative image depicting blood vessels with branch‐like protrusions and B) schematic depicting the regions of interest. Blood vessels were analyzed in an area up to 300 µm region (yellow/green) around the stroke core (red). C) Immunofluorescent staining of coronal brain sections with CD31. The dotted white line indicates the ischemic border zone (ibz). Scale bar: 100 µm. D) Analysis of vasculature density (vascular area fraction, number of branches, vessel length,) in the ischemic cortex. E) Immunofluorescent staining with GFAP. Scale bar: 50 µm. The ischemic border zone (ibz) and stroke core are shown to illustrate the stroke‐lesioned area. F) Quantification of GFAP immunoreactivity in the ischemic border zone. Dotted line = contralateral side. G) Immunofluorescent staining with IBA1. Scale bar: 50 µm. The ischemic border zone (ibz) and stroke core are shown to illustrate the stroke‐lesioned area. H) Quantification of IBA1 immunoreactivity in the ischemic border zone and in the stroke core. Dotted line = contralateral side. I) Representative histological overview of caspase‐3 and DAPI. Scalebar: 10 µm. J) Quantification of casp‐3 positive cells in the unaffected contralesional hemisphere, the ischemic border and the stroke core zone. Data is relative to all counted DAPI‐positive cells. Data are shown as mean distributions where the red dot represents the mean. Boxplots indicate the 25% to 75% quartiles of the data. For boxplots: each dot in the plots represents one animal. Significance of mean differences was assessed using an unpaired t‐test (acute vs. delayed. In total, *n* = 4 mice per group were used. Asterisks indicate significance: *p < 0.05, **p < 0.01, ***p < 0.001.

43 days after stroke induction, we applied CD31 immunostaining on coronal brain sections to visualize blood vessels and define the ischemic border zone, which extended up to 300 µm around the stroke core (**Figure** **2**A–C). We analyzed vascular area fraction (NPC_acute_: 15.2% NPC_delayed_: 17.7%, p = 0.3), vessel length (NPC_acute_: 27 ± 5.4, NPC_delayed_: 29 ± 1.9 mm mm^−2^, p = 0.4), and branch number (NPC_acute_: 628 ± 393, NPC_delayed_: 553 ± 98, mm^−2^, p = 0.7). We found no significant differences between the two experimental groups in the ischemic hemisphere (Figure 2D). Similarly, there were no differences in these vascular parameters between the groups on the contralateral side (Figure 2C; Figure S2A,B, Supporting Information).

We measured GFAP immunoreactivity in the ibz of both treatment groups to evaluate glial scar formation (Figure 2E). In both groups, the ibz displayed elevated signal intensity compared with the contralesional cortex (Figure 2F). Although GFAP immunoreactivity was higher in animals that received acute NPC treatment, this difference was not statistically significant (NPC_acute_: +242%, NPC_delayed_: +138%, p = 0.3). Histological analysis of the ipsilateral cortex revealed a general increase in Iba1 expression, a marker of microglia, with a pronounced signal in the stroke core (Figure 2G,H), relative to a corresponding region of interest in the contralateral hemisphere. Within the core region, Iba1 immunoreactivity was more strongly increased in animals given acute NPC treatment compared with those treated later; however, the difference did not reach statistical significance (**ibz**: NPC_acute_: +18.8%, NPC_delayed_: +8.4%, p = 0.34; **core**: NPC_acute_: +86.8%, NPC_delayed_: +13.5%, p = 0.11).

Apoptotic cells were investigated in the ischemic border zone and the stroke core using capsase‐3 antibodies (Figure 2I). Caspase‐3^+^ cells were found in the ischemic border zone (NPC_acute_: 9.4 ± 1.3, NPC_delayed_: 12.7 ± 1.2, p = 0.12) as well as in the stroke core (NPC_acute_: 5.8 ± 1.1, NPC_delayed_: 5 ± 1.1, p = 0.9), but barely in the uninjured contralesional side (Figure 2J). However, no significant differences in caspase‐3^+^ counts were found between acute and delayed treatment groups.

These results suggest that the timepoint of NPC transplantation does not affect vascular repair and maturation, axonal glial scarring or apoptosis.

### 2.3 Delayed Transplantation Enhances Early Proliferation Without Altering Graft Differentiation

**Figure 3:**
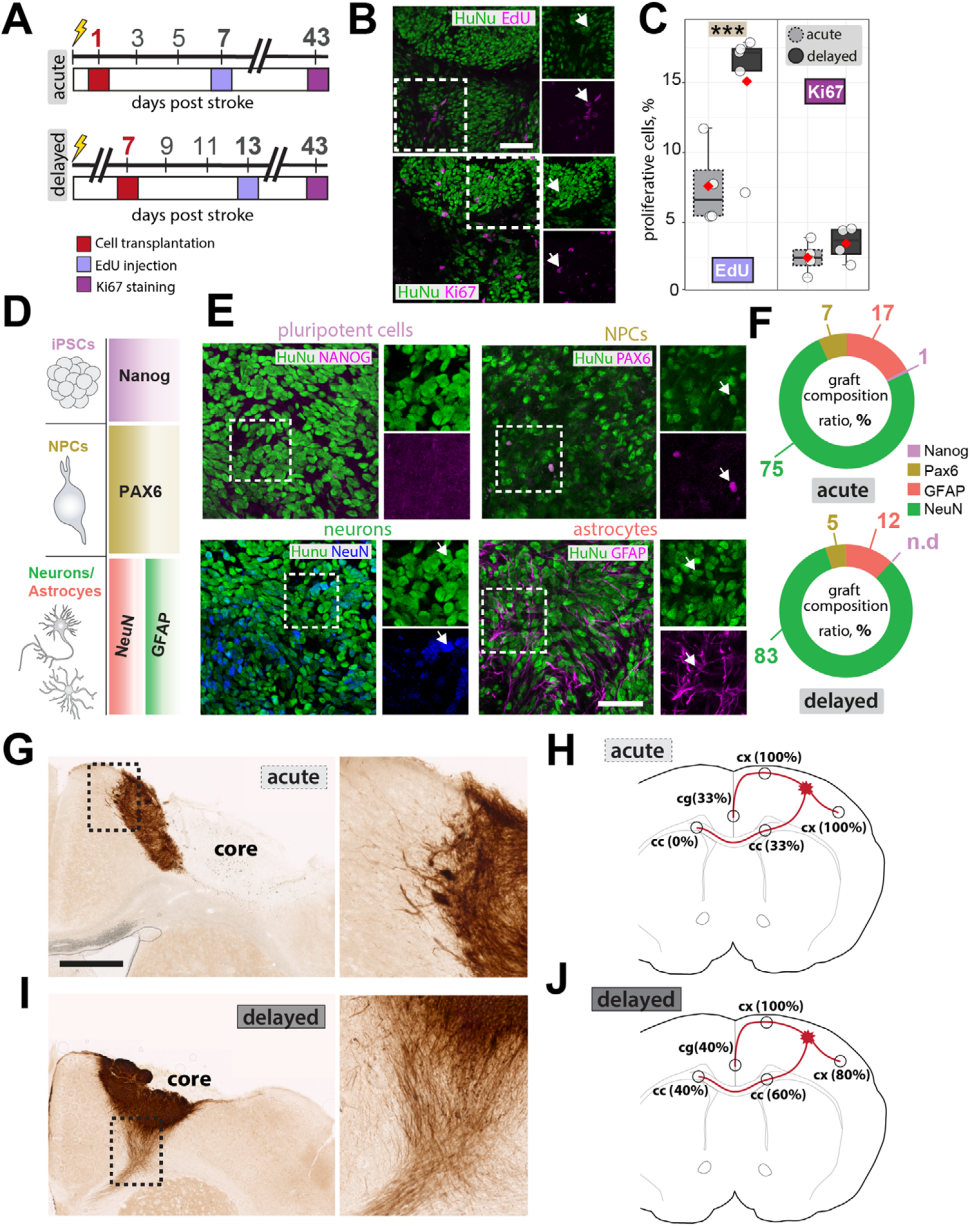
Quantification of graft composition after acute and delayed transplantation. A) Schematic timeline of proliferation assessment using EdU incorporation at 7 days post‐transplantation and Ki67 staining at 42 days (acute) and 35 days (delayed) post‐transplantation to track graft proliferation. B) Representative immunofluorescence images (scale bars: 50 µm) and C) quantification of EdU^+^ NPCs at seven days post‐transplantation and Ki67^+^ NPCs at 35 (delayed) and 42 (acute) days in both groups. D) Illustration showing phenotyping panel with pluripotency marker Nanog, NPC marker PAX6, neuronal marker NeuN, and astrocytic marker GFAP. E) Representative immunofluorescence images of grafted NPCs (HuNu+) at six weeks post‐transplantation. Scale bars: 50µm. F) Quantification of graft composition in acute vs. delayed transplantation groups. G) Representative images of hNCAM staining showing the ipsilesional cortex (acute). Scale bar: 50 µm. H) The percentage of acutely transplanted animals in which grafts extended neurites to various brain regions. I) Representative images of hNCAM staining showing the ipsilesional cortex (delayed). Scale bar: 50 µm. J) The percentage of delayed transplanted animals in which grafts extended neurites to various brain regions. Data are shown as mean distributions where the red dot represents the mean. In (C), a total of *n* = 9 animals were used (acute:4, delayed: 5); in (F, H, J) a total of *n* = 8 animals were used, with 4 animals per group. Boxplots indicate the 25% to 75% quartiles of the data. For boxplots: each dot in the plot represents one animal. Significance of mean differences was assessed using an unpaired t‐test (C, G). Statistical significance was set at *p < 0.05; **p < 0.01; ***p < 0.001.

We then aimed to determine whether the observed differences in bioluminescence signal were due to increased proliferation early after transplantation or at later stages. To investigate this, we injected the nucleotide analog EdU to label proliferating cells at 7 days post‐transplantation and conducted Ki67 staining after tissue collection (**Figure** **3**A,B). We detected a twofold increase in EdU proliferation within the graft in NPC_delayed_ compared to NPC_acute_ one week post transplantation (15.1% vs 7.6%; p < 0.001, Figure 3C). Notably, NPC proliferation levels were at similar low rates at 43 days after stroke (NPC_acute_: 2.5% Ki67^+^ cells vs NPC_delayed_: 3.5% Ki67^+^ cells, p > 0.05, Figure 3C).

Most transplanted NPCs differentiate into astrocytes and neurons within the first month, as shown in previous studies.^[^ ^12^ ^]^ To identify whether the transplantation timing after stroke can affect the graft composition, we phenotyped human nuclei (HuNu^+^) positive cell grafts for pluripotent stem cell (Nanog), NPC (Pax6), mature neuronal (NeuN), and astrocytic (GFAP) markers (Figure 3D,E). We observed that the majority of cell grafts differentiate into NeuN^+^ mature neurons (NPC_acute_: 75% vs NPC_delayed_: 83%), with only a small fraction of grafts expressing astrocytic GFAP^+^ (NPC_acute_: 17% vs NPC_delayed_: 12%), and Pax6 (NPC_acute_: 7% vs NPC_delayed_: 5%, Figure 3F). We additionally co‐stained with HuNu and Olig2 antibodies to determine oligodendrocyte differentiation of transplanted NPCs in the ischemic border zone and in the stroke core region (Figure S3A,B, Supporting Information). A total of 1.3% of cells were counted double‐positive for HuNu and Olig2 in the ischemic border zone in the NPC_acute_ group, while 0.95% of double‐positive cells were found in the NPC_delayed_ group (p = 0.23). In the stroke core, 1.3% of cells were double positive for HuNu and Olig2 in the NPC_acute_ group, whereas only 0.5% were HuNu/Olig2‐positive in the NPC_delayed_ group (p = 0.057). Overall, these findings suggest that delayed NPC transplantation promotes an initial surge in proliferation rates, possibly through a less hostile stroke environment. The timing of transplantation does not seem to affect overall differentiation patterns, as both acute and delayed grafts consistently maintain a similar neuronal‐to‐astrocytic ratio, with no detectable pluripotent residuals.

We then used neural cell adhesion molecule (hNCAM) immunostaining to visualize neurite outgrowth from the graft (Figure 3G,I). Neurites extended from the graft into multiple brain regions, including the contralateral hemisphere. Among animals receiving acute cell transplants, 33% exhibited axonal extensions to the ipsilateral corpus callosum (cc), while we did not find any neurites along the cc to the contralateral hemisphere (Figure 3H). In 33% of the animals that received acute NPC grafts, we observed axonal extensions to the cingulate cortex (Figure 3H). In the delayed transplantation group, 60% exhibited axonal extensions to the ipsilateral corpus callosum (cc), while 40% extended their neurites along the cc to the contralateral hemisphere (Figure 3J). In 40% of the animals that received acute NPC grafts, we observed axonal extensions to the cingulate cortex (Figure 3J). Notably, in 100% of all transplanted animals, cells extended their neurites along the ipsilateral cortex to the primary and secondary motor cortex.

## 3 Discussion

In this study, we show that delayed (7 dpi) transplantation of NPCs significantly improves graft proliferation and survival compared to acute (1 dpi) transplantation in a photothrombotic stroke model.

In the acute phase following stroke, the cerebral microenvironment is characterized by highly inflammatory responses, oxidative stress, and a disrupted blood brain barrier (BBB), thus creating a hostile setting for cell therapy. As early as 24 h post‐stroke, reactive oxygen species (ROS) and a plethora of inflammatory mediators (TNF‐α, IL‐1β, IL‐6, etc.) compromise the BBB, leading to infiltration of immune cells (neutrophils, macrophages, and T cells) into the brain parenchyma.^[^ ^13^ ^]^ At the same time, matrix metalloproteinases (MMPs) degrade perivascular basement membranes and tight junction proteins, further increasing BBB permeability, enabling the entrance of toxic substances.^[^ ^14^, ^15^ ^]^ These factors not only induce direct cellular damage but also might hinder the integration and differentiation of newly transplanted cells. After the early activation of the immune system, the inflammatory milieu partially subsides,^[^ ^16^ ^]^ the BBB regains integrity,^[^ ^17^, ^18^ ^]^ and oxidative stress levels diminish.^[^ ^19^ ^]^ Thus, the subacute phase may offer a more favorable environment for the survival and engraftment of transplanted NPCs, compared to the highly hostile conditions that prevail during the acute phase.^[^ ^5^ ^]^ Future studies should explore the mechanisms underlying the enhanced survival observed in grafts transplanted at delayed timepoints (7dpi), particularly by investigating how inflammatory modulation and niche adaptation contribute to host–graft integration. Additionally, cell priming strategies, such as exposing NPCs to hypoxia or hypothermia, pre‐conditioning cells with growth factors, gene‐editing to promote the expression of protective genes, modulating the immune response, or providing a supportive scaffold, should be explored to determine whether these approaches improve graft survival, engraftment, and proliferation within the subacute stroke environment. Indeed, these strategies have been shown to trigger adaptive responses in cells, such as increased growth factor secretion and improved stress tolerance, which may enhance NPC viability and integration in the injured brain. Understanding the molecular mechanisms behind these approaches could help optimize cell‐based therapies for better functional recovery after stroke.

Previous studies have reported that delayed transplantation following lesion ameliorated graft survival rates. Embryonic motor cortical neuron grafts showed significantly enhanced vascularization, survival, and transplant‐to‐host projections when transplanted 1 week after injury, compared to earlier transplantation timepoints.^[^ ^20^ ^]^ And delivery of bone marrow‐derived MSCs between 2‐ and 7‐days post‐stroke significantly increased outcome compared to administration 24 h after injury.^[^ ^21^ ^]^ In our study, we found no evidence that the timing of NPC therapy affected vascular repair 6 weeks after stroke induction. However, a recent study from our lab demonstrates that NPC treatment (regardless of transplantation timing) can significantly increase blood vessel density in the ischemic core compared to a vehicle treatment.^[^ ^12^ ^]^ NPC grafts are suggested to secrete angiogenic factors, such as VEGF, ANG1, FGF2, PDGFA and HIF‐1a, that promote vascular repair and vessel maturation, thereby supporting tissue regeneration while reducing the risk of vascular leakage and bleeding.^[^ ^3^, ^12^, ^22^, ^23^, ^24^ ^]^ Previous research has demonstrated that cortical infarcts undergo a relatively brief period of pronounced vascular structural plasticity during the first two weeks after stroke.^[^ ^25^ ^]^ Within this critical window, newly formed capillary connections help restore blood flow in peri‐infarct tissue, thereby alleviating local ischemia, supporting synaptic reorganization, and helping functional recovery. In our study, we transplanted NPCs on days 1 and 7 post‐stroke to align with this critical phase. By delivering the grafted cells during a phase when endogenous reparative processes are most active, we aimed to enhance the synergy between the pro‐angiogenic cues released by grafted NPCs and the host's own effort to promote vascular repair.

We also assessed the amount of glial scarring between the two transplantation groups and found no significant difference. Neuroinflammation contributes to a hostile environment and triggers glial cell activation, playing a dual role in repair processes. Astroglial scarring can aid in tissue stabilization, while microglial activation is associated with pro‐inflammatory responses and tissue injury.^[^ ^26^ ^]^ Although significant downregulation of GFAP expression after stem cell‐based therapy has been reported in MSC studies,^[^ ^27^, ^28^ ^]^ NPCs may exhibit a different mechanism of action, potentially relying more on neuronal differentiation and integration rather than dampening astrocyte reactivity. For decades, the prevailing dogma held that glial scarring is a key inhibitor of CNS regeneration;^[^ ^29^, ^30^, ^31^ ^]^ however, more recent evidence suggests that astroglial scars can also support axon regeneration in spinal cord injury.^[^ ^32^ ^]^ In our experiments, we observed neurite extension from the graft to distinct brain regions, indicating that transplanted cells may navigate or even modify the glial scar. Conversely, the glial scar may provide structural and chemical signals essential for the functional integration of cell grafts.^[^ ^33^ ^]^ The lack of difference in glial scarring between the acute and delayed groups points to the complexity of post‐stroke repair, where timing may not strongly influence certain facets of inflammatory scarring but could still affect other repair processes.

Interestingly, we did not observe differences in the graft composition. While few previous studies have suggested that hypoxic changes can alter the glia vs neuron fate commitment in NPC to the glial phenotype,^[^ ^34^, ^35^, ^36^ ^]^ we did not observe a significant change in the graft composition. Potentially, these findings may indicate that the stroke microenvironment do not exhibit strong differences in O₂ availability at 1 and 7 days post injury, but instead reflects a reduced inflammatory state, which has been observed before in stroke patients.^[^ ^37^ ^]^

While our results show that the overall number of NPCs increases over six weeks when cell grafts are transplanted one week after injury, it is important to note that this enhanced bioluminescent signal does not indicate a potential tumor formation. In a previous study, we found no histological evidence of tumorigenesis and instead observed that most grafted NPCs differentiated into neurons, as evidenced by the high positivity for the neuronal markers NeuN, MAP2, and βIII‐tubulin.^[^ ^4^ ^]^ The broader extension of neurites from delayed NPC grafts into distinct brain regions could be due to the more permissive environment during the subacute phase, where reduced inflammation and a partially restored BBB may allow for better axon outgrowth and host–graft integration. Additionally, NPCs might secrete neurotrophic factors and guidance cues more effectively when the environment is less hostile. Several studies have shown that post‐stroke motor improvement correlates with increased neuronal connectivity and axonal sprouting.^[^ ^34^, ^38^, ^39^ ^]^ hNSC transplantation into the ischemic brain enhanced axonal growth, upregulated genes involved in neurogenesis and trophic support, and improved motor function.^[^ ^39^ ^]^ Similarly, hiPSC‐derived glial progenitors transplanted in the subacute phase after white matter stroke promoted axonal sprouting and enhanced motor and cognitive recovery.^[^ ^34^ ^]^ Here, we also observed graft‐derived axonal sprouting on the contralesional side, raising the possibility that NPCs contribute to the formation of alternative descending motor pathways.^[^ ^40^, ^41^ ^]^ Such pathways may originate from the contralateral motor cortex and relay through subcortical structures like the red nucleus or pontine formation, or they may involve direct compensatory corticospinal projections to support functional recovery.^[^ ^42^, ^43^, ^44^ ^]^ The ability to regenerate axonal projections after stroke holds promise for advancing clinical therapies. To fully understand the therapeutic potential of NPCs, it will be important to define the specific molecular programs they engage in vivo and understand how these influence their integration, differentiation, and functional impact after transplantation.

From a clinical standpoint, delayed transplantation offers several advantages, including improved patient stabilization, a potentially better prediction of stroke injury progression, and the opportunity to extend the currently limited 4–24‐h therapeutic window.

Overall, our findings suggest that delaying NPC transplantation by one week may enhance the therapeutic potential of NPC‐based therapies in stroke and potentially other related neurological disorders.

## 4 Experimental Section

### Animals

All animal experiments were conducted at the Laboratory Animal Services Center (LASC) in Schlieren in compliance with local regulations for animal research and approved by the Veterinary Office in Zurich, Switzerland. A total of 10 (four male and six female) adult genetically immunodeficient Rag2^−/−^ mice (between 10 and 12 weeks, 20–30 g) were used for this study. Graft proliferation was assessed in 9 animals(1 excluded due to unsuccessful EdU labeling), and 8 were used for stroke size, vascular repair, gliosis, and graft composition analyses (2 excluded due to incomplete stroke tissue processing). The group sizes were determined by power calculations and prior studies,^[^ ^4^, ^12^, ^45^, ^46^ ^]^ aiming for 80% power to detect large effects (Cohen's d = 1–2) at α = 0.05. Mice were housed at the LASC in top‐filter laboratory cages under OHB conditions in a temperature‐ and humidity‐controlled environment with a 12/12‐h light/dark cycle (lights on from 6:00 a.m. to 6:00 p.m.). Animals were housed in groups of two to four per cage and had ad libitum access to standard diet food pellets and water.

Both sexes were used and analyzed for this study, the statistical analyses did not reveal any significant sex‐related differences in the measured parameters.

### Cell Culture

For the cell‐based stroke therapy, a chemically defined, xeno‐free differentiation protocol was developed to generate neural progenitor cells (NPCs) from human iPSCs.^[^ ^4^ ^]^ Briefly, iPSCs (80 000 per well) were seeded on vitronectin‐coated 12‐well plates (StemMACS iPS‐Brew XF supplemented with 2 εm Thiazovivin) and incubated overnight. After replacing the medium, neural differentiation started the following day with a standard induction medium (50% DMEM/F12, 50% Neurobasal medium, 1 × N2‐supplement, 1 × B27‐supplement, 1 × Glutamax, 10 ng mL^−1^ hLIF, 4 µm CHIR99021, 3 µm SB431542, 2 µm Dorsomorphin, 0.1 µm Compound E). After 24 h, the medium was refreshed and, on day 2, switched to a modified neural induction medium (50% DMEM/F12, 50% Neurobasal medium, 1 × N2‐supplement, 1 × B27‐supplement, 1 × Glutamax, 10 ng mL^−1^ hLIF, 4 µm CHIR99021, 3 µm SB431542, 0.1 µm Compound E). On day 6, cells were transferred to pLO/L521‐coated plates in neural stem cell maintenance medium (NSMM: 50% DMEM/F12, 50% Neurobasal medium, 1 × N2‐supplement, 1 × B27‐supplement, 1 × Glutamax, 10 ng/ml hLIF, 4 µm CHIR99021, 3 µm SB431542), passaged at 80–100% confluency, and supplemented from passage two onward with 5 ng ml^−1^ FGF2. During the first six passages, 2 εm Thiazovivin was added after splitting. Gene and protein expression analysis confirmed the stable upregulation of NPC markers (Pax6, Sox1, Nestin) and the downregulation of pluripotency markers (Oct4) over 15 passages, indicating successful differentiation. Neural progenitors spontaneously differentiated into β‐III‐Tubulin‐ and MAP2‐positive neurons and S100B‐positive astrocytes when growth factors and small molecules were withdrawn. Before transplantation, NPCs were transduced with a lentiviral vector encoding a bioluminescent (red firefly luciferase) and fluorescent (eGFP) reporter, as previously described,^[^ ^4^ ^]^ enabling in vivo tracking of transplanted cells.

### Photothrombotic Stroke Induction

Photothrombotic stroke was induced as previously described.^[^ ^17^, ^47^, ^48^, ^49^ ^]^ Anesthesia was initiated with 4% isoflurane in oxygen and maintained at 1–2% using a self‐fabricated face mask. Mice were positioned in a stereotactic frame (David Kopf Instruments), and body temperature was maintained at 36–37 °C using a heating pad. Anesthesia depth was confirmed by the absence of a toe‐pinch reflex, and eye lubricant (Vitamin A, Bausch & Lomb) was applied to prevent corneal drying. The head was shaved, and Emla Creme 5% (lidocaine/prilocaine) was applied to the scalp and ears before inserting the ear bars for skull fixation. A ∼1 cm midline incision was made to expose bregma and lambda, and the periosteum of the lower right hemisphere was scraped off. The exact stroke induction site [−2.5 to +2.5 mm medial/lateral and 0 to +3 mm from bregma] was identified under an Olympus SZ61 surgical microscope and marked. Rose Bengal (15 mg mL^−1^ in 0.9% NaCl) was injected intraperitoneally (10 µl g^−1^ body weight) 5 min before illumination to allow systemic circulation. The marked cortical region was illuminated for 10 min using an Olympus KL1500LCD cold light source (150W, 3000K, 4mm × 3mm surface area). After illumination, mice were placed in a recovery cage, and stroke induction was confirmed 2 h post‐surgery using laser Doppler imaging (LDI). The incision was sutured using a Braun surgical suture kit, disinfected with Betadine, and animals were monitored under standard post‐operative care.

### NPC Transplantation

Transplantation of neural progenitor cells (NPCs) was performed as previously described.^[^ ^12^, ^46^ ^]^ The surgical procedure followed the photothrombotic stroke model up to the point of illumination. Animals were divided into three groups: NPC transplantation 1 day post‐stroke (acute), NPC transplantation 7 days post‐stroke (delayed), and a vehicle control group.

After identifying bregma, the stereotactic device was adjusted to the injection site coordinates [AP: +0.5 mm, ML: +1.5 mm, DV: −0.6 mm relative to bregma]. A small ∼0.8 mm hole was drilled into the skull, ensuring penetration through the cortical bone. A 10 µl Hamilton syringe fitted with a 30G needle containing 2.5 µl of NPC suspension (1 × 10⁵ cells µl^−1^) was mounted onto the stereotactic device and positioned above the injection site. The needle was slowly lowered to the calculated depth, with an additional 0.05 mm penetration to create a small cavity, preventing cell suspension spillover. Once retracted to the target depth, NPCs were injected at a steady rate of 2 nl/s into the brain parenchyma. After injection, the needle remained in place for 5 min to allow cell settlement before being withdrawn slowly. The cavity was sealed with Histoacryl, and once hardened, the skin was sutured. Animals were returned to an empty cage and monitored under standard post‐operative care.

### Bioluminescent Imaging (BLI)

Animals were imaged using the IVIS Lumina III In Vivo Imaging System, with the head region carefully shaved using an electric razor to ensure optimal signal detection. Mice received an intraperitoneal injection of D‐luciferin (300 mg kg^−1^ body weight) dissolved in PBS, which was sterile‐filtered through a 0.22 µm syringe filter. Bioluminescence images were acquired at multiple time points (1, 3, 7, 14, 21, 28, 35, and 43, days post‐injury) to monitor transplanted cell survival and distribution.

Imaging data were analyzed using Living Image software (V 4.7.3). A size‐constant region of interest (ROI) (height, width = 1.8 cm) was positioned directly over the color‐coded signal between the ears and nose of each animal. Two additional ROIs were placed: one at the posterior body region to quantify background signal and another in a randomly selected area proximal to the body to measure image noise. The background‐subtracted signal was then used to calculate the maximum photon emission (PEmax). Bioluminescence signals were measured in photons per second per square centimeter per steradian (ph/s/cm^2^/sr). Data was recorded in Microsoft Excel and visualized using R software for statistical analysis.

### Tissue Processing and Immunohistochemistry

Animals were transcardially perfused with Ringer solution (0.9% NaCl). Brain tissue was then extracted and immediately frozen for immunohistochemistry. Animals were perfused first with Ringer solution, followed by 4% PFA, and brains were subsequently incubated in 4% PFA for 6 h. Coronal brain sections (40 µm) were prepared using a Thermo Scientific HM 450 microtome, washed in 0.1 m phosphate‐buffered saline (PBS), and blocked for 1 h at room temperature in 500 µl of blocking buffer containing 5% donkey serum diluted in 1× PBS + 0.1% Triton X‐100. Sections were then incubated overnight at 4 °C on a Unimax 1010 shaker (∼90 rpm) with the primary antibodies (HuNu, 1:200, EMD Millipore; CD31, 1:50, BD Biosciences; Fibrinogen, 1:100, Abcam; GFAP, 1:200, R&D Systems; Casp‐3, 1:200, Cell Signaling; hNanog, 1:30, R&D Systems, Pax6, 1:40, R&D Systems; NeuN, 1:400, Invitrogen; Ki67, 1:200, Invitrogen). The following day, sections were washed in PBS and incubated with the appropriate secondary antibody for 2 h at room temperature, followed by staining with DAPI (Sigma, 1:2000 in 0.1 m PBS). Finally, sections were mounted onto Superfrost Plus microscope slides and coverslipped using Mowiol mounting solution.

To further evaluate cell proliferation 1 week after cell transplantation, 5‐ethynyl‐2′‐deoxyuridine (EdU) labeling was performed. EdU was dissolved in sterile PBS at a concentration of 5 mg mL^−1^ and administered via intraperitoneal injection at 50 mg kg^−1^ body weight 7 days after transplantation. Brain sections were incubated for 30 min with a reaction mix buffer (1× per well: 325 µl ddH₂O, 25 µl 2 m Tris, 100 µl 10mm CuSO₄, 0.1 µl 10mm AlexaFluor 647, 100 µl 500mm ascorbic acid). Sections were then washed with 0.1 m PBS, counterstained with DAPI, mounted onto Superfrost Plus microscope slides, and coverslipped with Mowiol solution.

### Histological Quantification of Vasculature, Glial Scar Formation, and Axonal Sprouting

All analyses were performed in the peri‐infarct region, referred to as the ischemic border zone (ibz), which extends up to 300 µm from the stroke core. Post‐ischemic angiogenesis was evaluated using FIJI (ImageJ, version 2.1.0/1.53c) and a previously developed script that automatically calculates 1) vascular density, 2) the number of branches and junctions, and 3) the length of blood vessels.^[^ ^50^ ^]^ To assess glial scar formation, three brain sections per animal were immunostained for GFAP (astrocyte marker). The resulting images were converted to 8‐bit format and thresholded based on mean gray values obtained from regions of interest (ROIs) in the unaffected contralateral cortex, producing a binary image. The cumulative area of reactive gliosis in the ibz was then quantified.

To visualize neurites extending from the transplanted cells, 3,3′‐Diaminobenzidine Tetrahydrochloride (DAB) staining was performed for a human‐specific neural cell adhesion molecule (hNCAM). Coronal sections were imaged on the Zeiss Axio Scan.Z1 slide scanner and later processed using Fiji.

### Statistical Analysis

Statistical analysis was performed using RStudio (Version 4.04). Sample sizes were designed with adequate power in line with previous studies from the group and relevant literature. Data normality was assessed using the Shapiro‐Wilk test. Post hoc multiple comparisons were corrected using the Bonferroni method. For bioluminescence imaging analysis, signal intensity was compared day‐wise using an unpaired Mann–Whitney *U *test. Normally distributed data was tested for differences with an unpaired two‐sided t‐test to compare changes between two treatment groups (i.e differences between ipsi‐ and contralesional sides, or between treatment groups). Blood flow measurements obtained by Laser doppler were compared between healthy and injured hemisphere using an unpaired Mann–Whitney *U *test. Box plots display median, interquartile range (IQR), and individual data points. Line graphs are expressed as mean ±SEM. Statistical significance was set at p < 0.05.

### AI Assistance Disclosure

Grammar and typos were corrected with the assistance of ChatGPT V 4o (OpenAI). No content generation or data analysis was conducted using AI.

### Ethics Declaration

All in vivo experiments were performed at the Laboratory Animal Services Center (LASC) in Schlieren, Switzerland according to the local guidelines for animal experiments and were approved by the Veterinary Office of the Canton Zurich in Switzerland (Protocol number ZH209/2019 and ZH110/2023).

## Conflict of Interest

The authors declare no conflict of interest.

## Author Contributions

R.Z.W., C.T., and R.R. contributed to overall project design. R.Z.W., N.H.R., L.N., B.A.B., C.T., and R.R. conducted and analyzed experiments. M.G. provided iPS cell line. R.Z.W. and R.R. made figures. CT, RR supervised the study. R.Z.W., L.N., C.T., and R.R. wrote and edited the manuscript with input from all authors. All authors read and approved the final manuscript.

## Supporting information



Supporting Information

Supplemental Dataset 1

## Data Availability

The data that support the findings of this study are available in the supplementary material of this article.
