# Supplementary material for: Delayed Transplantation of Neural Stem Cells Improves Initial Graft Survival after Stroke
Source: Adv Sci (Weinh). 2025 May 23;12(29):e04154. doi: 10.1002/advs.202504154 (PMC12362810; doi:10.1002/advs.202504154)
Supplement: Supplementary file 1 — Supporting Information [file ADVS-12-e04154-s002.docx]

**SUPPLEMENTAL FIGURES**

**Delayed Transplantation of Neural Stem Cells Improves Initial Graft Survival After Stroke**

*Rebecca Z Weber^1,2^, Nora H Rentsch^1,2^, Beatriz Achón Buil^1,2^, Melanie Generali^1^, Lina R. Nih^3^, Christian Tackenberg^1,2,^****^*^*** *Ruslan Rust^4,5.^****^*^***

1 Institute for Regenerative Medicine, University of Zurich, Schlieren, 8952, Switzerland,

2 Neuroscience Center Zurich, ETH Zurich and University of Zurich, Zurich, 8057, Switzerland

3 Department of Brain Health, Kirk Kerkorian School of Medicine, University of Nevada Las Vegas, NV 89154, USA

4 Department of Physiology and Neuroscience, University of Southern California, Los Angeles, CA 90033, USA

5 Zilkha Neurogenetic Institute, Keck School of Medicine, University of Southern California, Los Angeles, CA 90033, USA

* co-corresponding

**Correspondence:**

Ruslan Rust, Ph.D.
Assistant Professor
Assistant Director of the Stem Cell Unit
The Zilkha Neurogenetic Institute
Department of Physiology and Neuroscience
Keck School of Medicine of the University of Southern California
1501 San Pablo Street, Room 341
Los Angeles, CA 90033
email: [rrust@usc.edu](mailto:rrust@usc.edu)
ORCID: 0000-0003-3376-3453

Christian Tackenberg, Ph.D.
Scientific Head of Division
Institute for Regenerative Medicine • IREM
University of Zurich
Wagistrasse 12
8952 Schlieren, Switzerland
[christian.tackenberg@irem.uzh.ch](mailto:christian.tackenberg@irem.uzh.ch)
ORCID: 0000-0002-0019-3055


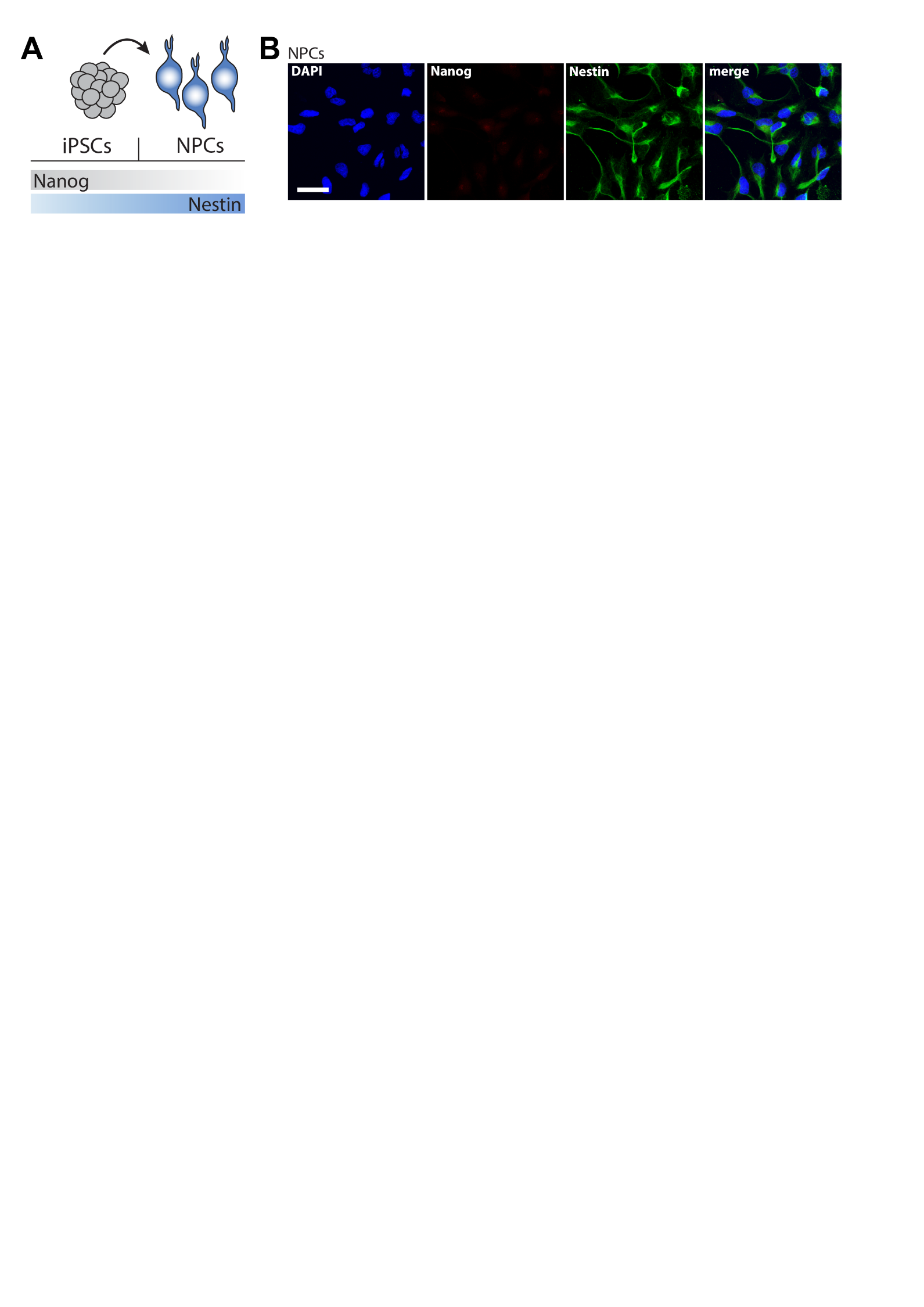


**Suppl. Figure 1: Generation of hiPSC-NPCs.** (A) Generation of neural progenitor cells (NPCs) derived from human induced pluripotent stem cells (hiPSCs). (B) NPCs (passage 7) stained for DAPI, Nanog, iPSC marker, and Nestin, a NPC marker. Scale bar: 10um.


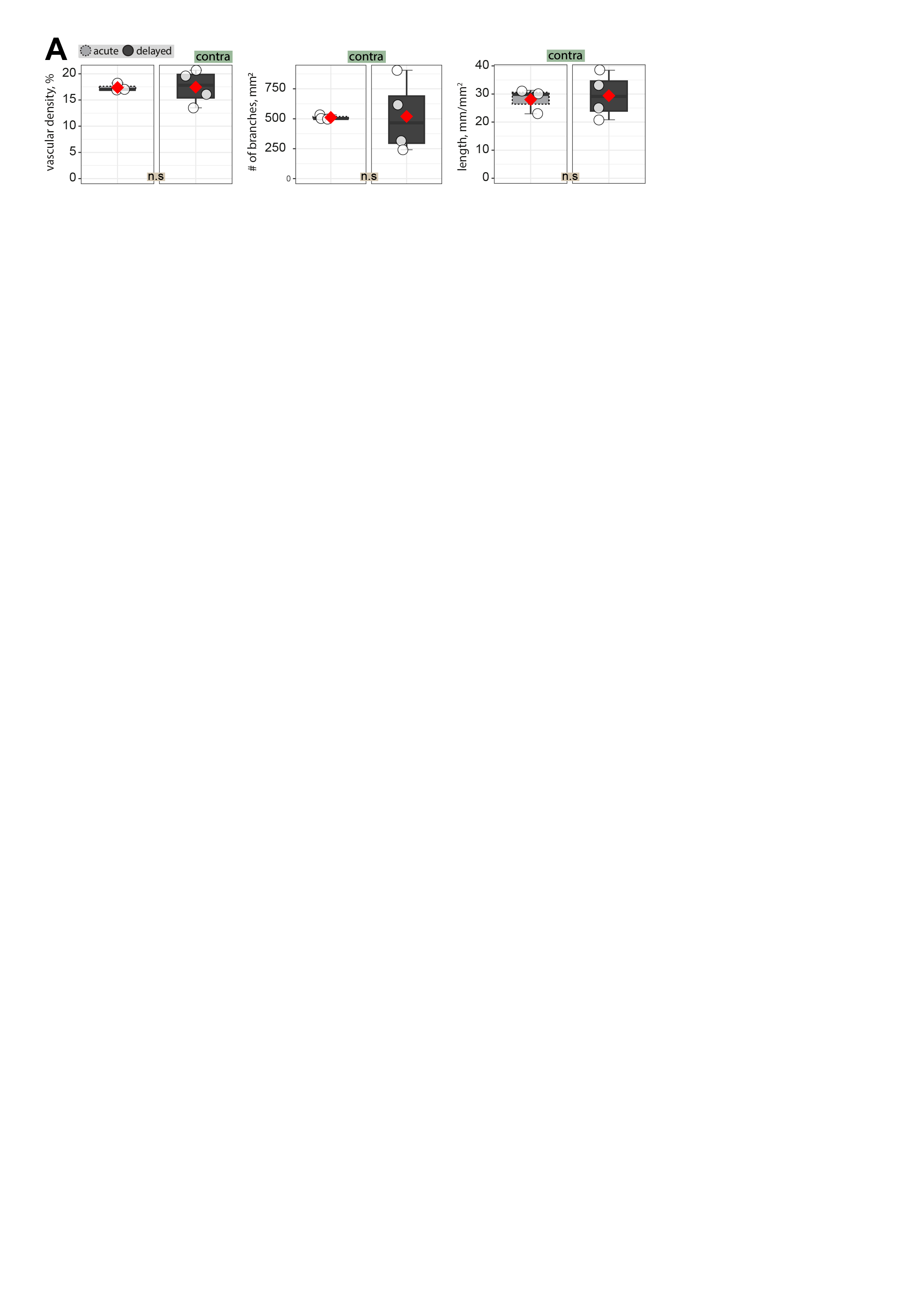


**Suppl. Figure 2: Vascular changes after stroke.** (A) Quantification of vascular density, including area, branch number, and length, in the intact brain 43 days after stroke. Scale bar: 100um. Data are shown as mean distributions where the red dot represents the mean. Boxplots indicate the 25% to 75% quartiles of the data. For boxplots: each dot in the plots represents one animal. Significance of mean differences was assessed using an unpaired t-test (acute vs. delayed). In B, n=4 (acute) and n=4 (delayed) mice per group were used.


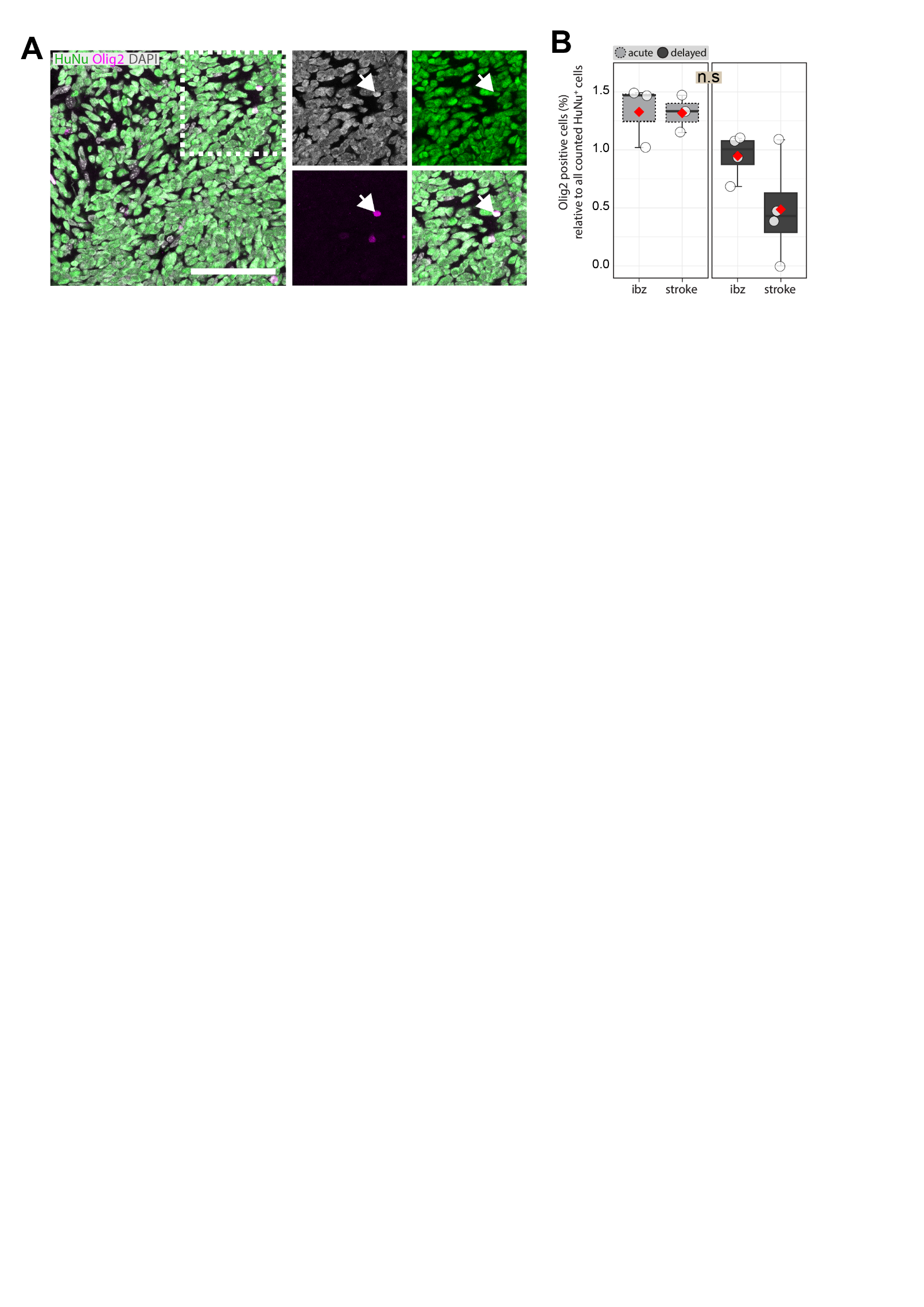


**Suppl. Figure 3: Fate of transplanted NPCs 35 days post-transplantation**. (A) Immunofluorescent staining of a coronal brain section with Olig2, HuNu and DAPI. (B) Quantification of Olig2/HuNu^+^ cell count relative to all counted HuNu^+^ cells. Scale bar = 50µm. Data are shown as mean distributions where the red dot represents the mean. Boxplots indicate the 25% to 75% quartiles of the data. Each dot in the plot represents one animal. Significance of mean differences was assessed using an unpaired t-test (acute vs. delayed). In B, n=4 (acute) and n=4 (delayed) mice per group were used.
